# Panniculitis in dermatomyositis: A systematic review of the clinicopathologic features

**DOI:** 10.1016/j.jdin.2024.03.004

**Published:** 2024-03-22

**Authors:** Jonathan D. Ho, Trimane McKenzie

**Affiliations:** a Division of Dermatology, Department of Medicine, The University of the West Indies, Mona Campus, Kingston, Jamaica; b Department of Pathology, The University of the West Indies, Mona Campus, Kingston, Jamaica

**Keywords:** autoimmune, autoimmune connective tissue disease, dermatomyositis, dermatopathology, lupus, panniculitis

## Abstract

**Background:**

Panniculitis in patients with dermatomyositis (PDMS) is rare.

**Objectives:**

Assess the clinicopathologic features described for PDMS.

**Methods:**

A systematic review of the PubMed/MEDLINE database was performed. We included cases with dermatomyositis (DMS) and confirmed panniculitis (histology/imaging). Extracted data included demographics, global region of origin, comorbidities, hallmark/nonhallmark features, muscle disease, panniculitis onset relative to classical DMS, symptomatology, physical findings, triggers, autoantibodies, extracutaneous involvement, histopathology, treatment, and prognosis.

**Results:**

Fifty-eight studies were included (91 patients). PDMS primarily occurred in relatively young women (mean age 35 years, female to male ratio 4.4:1). Adults predominated (adult:juvenile ratio 2.6:1). All had hallmark DMS features. A minority had nonhallmark findings. <1/4 reported extracutaneous involvement (pulmonary complications predominated). Cancer-associated disease was uncommon (7.4%). Panniculitis occurred before/simultaneously/after features of classical DMS. The clinical and histopathologic findings mimic lupus panniculitis, although lipoatrophy was uncommon (12.7%). A lobular/lobular predominant, lympoplasmacytic panniculitis was most common. Treatment regimens included combinations of systemic steroids/traditional steroid-sparers/Janus kinase inhibitors/biologics. The majority had a good/complete response (67.3%), but recalcitrant disease (22.4%) was reported.

**Limitations:**

Retrospective nature, inconsistent reporting of parameters, lack of longitudinal data.

**Conclusions:**

PDMS primarily occurs in females, mimics lupus panniculitis, and responds to therapy.

**Capsule Summary**

• We detail the clinicopathologic features of panniculitis occurring in patients with dermatomyositis.
• Increasing recognition of this entity by describing the demographics, clinical/histologic diagnosis, similarities with lupus panniculitis, utilized therapies, and outcomes of dermatomyositis-associated panniculitis.

## Introduction

The autoimmune connective tissue diseases (AICTDs) have many cutaneous manifestations. Although well documented in lupus erythematosus, panniculitis in patients with dermatomyositis (PDMS) is less often appreciated.^1^ We performed a systematic review of PDMS.

## Methods

A systematic review was performed using the Preferred Reporting Items for Systematic Reviews and Meta-Analyses guidelines.^2^ Literature search was completed on May 27, 2023 utilizing the PubMed/MEDLINE database. Keywords were “dermatomyositis” AND “panniculitis.” We included all publication types in any language (Google Translate). Inclusion required clinical features consistent with dermatomyositis (DMS) and confirmed panniculitis (histopathology/imaging). Reviews without primary data were excluded. Where abstracts lacked inclusion/exclusion information, full-texts were examined. Both authors independently screened titles/abstracts and articles for full-text reading. Thereafter, studies were included/excluded by consensus. Variables included demographics, global region of origin, comorbidities, medication history, juvenile/adult dermatomyositis (JDMS/ADMS), hallmark/nonhallmark features, muscle disease (clinical/laboratory/imaging evidence), panniculitis onset relative to classical DMS features, symptomatology, physical findings, triggers, autoantibodies, extracutaneous involvement, histopathology, treatment, and prognosis. Treatment was designated “specific” if an agent was added to general dermatomyositis (GDMS) treatment/specifically used for panniculitis control. Dose escalation of a preexisting regimen was not considered specific. Prognosis was assigned complete/marked, partial, or poor, as described. Descriptive statistics were generated (GoogleSheets and GNU project statistical package for the social sciences [Free Software Foundation] V.8.5). Association was examined using χ^2^ tests (categorical) and *t* tests (comparison of means). If specific data were unavailable, it was labeled missing. Only valid data points were utilized to calculate frequencies/averages/proportions.

## Results

### Selection

**Fig 1:**
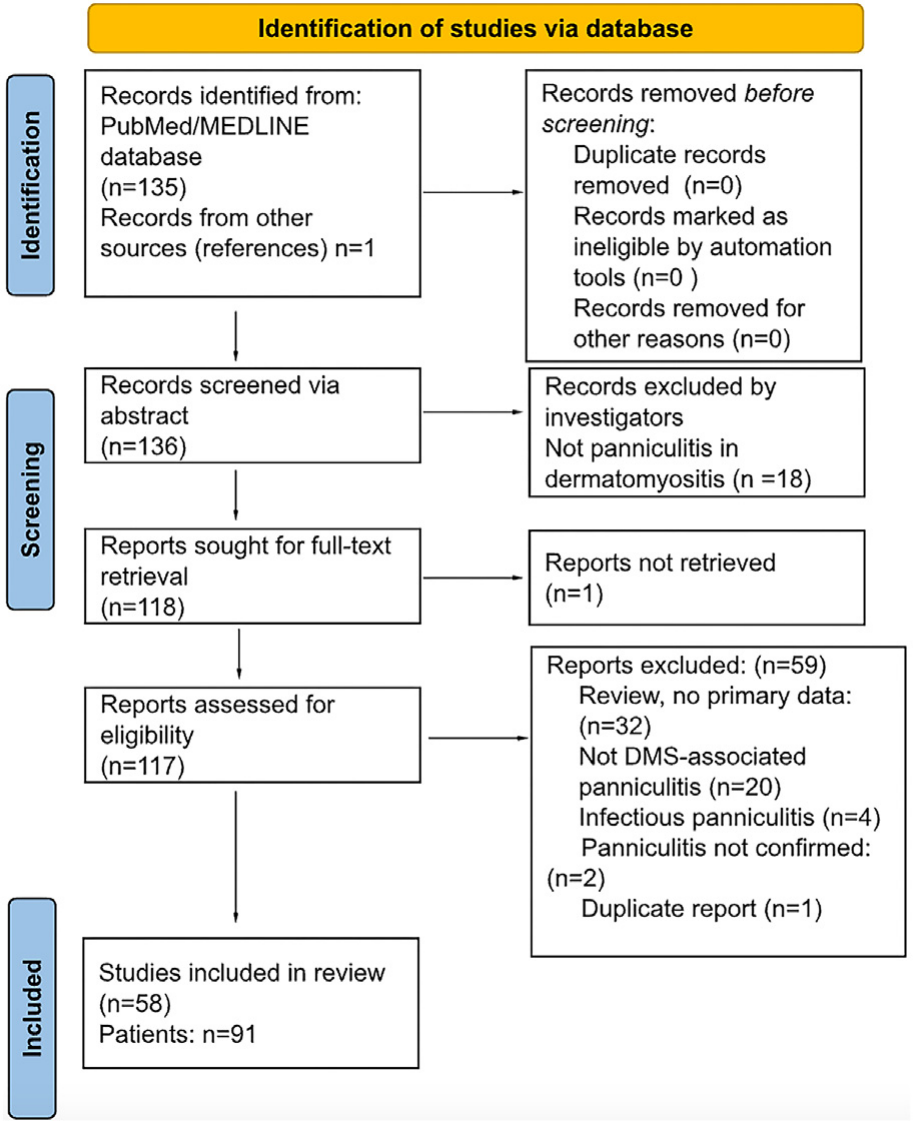
Preferred Reporting Items for Systematic Reviews and Meta-Analyses chart for dermatomyositis-associated panniculitis. *DMS*, Dermatomyositis.

Search strategy produced 135 citations (Fig 1). Reference review added 1 study. Abstract/title screening excluded 18 articles; 118 full-texts were evaluated. One study was inaccessible despite numerous purchase attempts. Fifty-nine further studies were excluded; 58 studies were included, providing data on 91 cases.^3^, ^4^, ^5^, ^6^, ^7^, ^8^, ^9^, ^10^, ^11^, ^12^, ^13^, ^14^, ^15^, ^16^, ^17^, ^18^, ^19^, ^20^, ^21^, ^22^, ^23^, ^24^, ^25^, ^26^, ^27^, ^28^, ^29^, ^30^, ^31^, ^32^, ^33^, ^34^, ^35^, ^36^, ^37^, ^38^, ^39^, ^40^, ^41^, ^42^, ^43^, ^44^, ^45^, ^46^, ^47^, ^48^, ^49^, ^50^, ^51^, ^52^, ^53^, ^54^, ^55^, ^56^, ^57^, ^58^, ^59^, ^60^

### Demographic and general medical data

Of 91 patients, 74 were female and 17 male (F:M ratio of 4.4:1). Specific age was available for 74, range = 1 to 83 years; mean age of 35 years (SD ± 22.1). Mean age in ADMS/JDMS was 45.3 (SD ± 17.1) and 9 (SD ± 5.43) years respectively. Regarding global region of origin, 35.2% (*n* = 32) originated from Asia, 24.2% (*n* = 22) Europe, 11% (*n* = 10) North America, 4.4% (*n* = 4) South America, and 2.2% (*n* = 2) each from Africa and Oceania; 20.9% (*n* = 19) reported from multiple locations. Where available (*n* = 33), race included 75.8% Asian (*n* = 25), 21.2% Caucasian (*n* = 7), and 3% Black (*n* = 1). Twelve cases were diagnosed via imaging (magnetic resonance imaging/ultrasound/computed tomography).^4^^,^^22^^,^^28^ Preexisting medical illness was reported in 6.6% (*n* = 6); hypertension (*n* = 1), obesity (*n* = 2), inflammatory bowel disease (*n* = 1), dyslipidemia (*n* = 1, concomitant obesity), lupus erythematosus (*n* = 1), and acne vulgaris (*n* = 1). Four reports detailed pre-DMS medication usage (minocycline, amlodipine, and mesalazine and methotrexate). Overall, PDMS was most commonly reported in relatively young females from a variety of geographic locations and no comorbid disease/drug history.

### GDMS features

**Fig 2:**
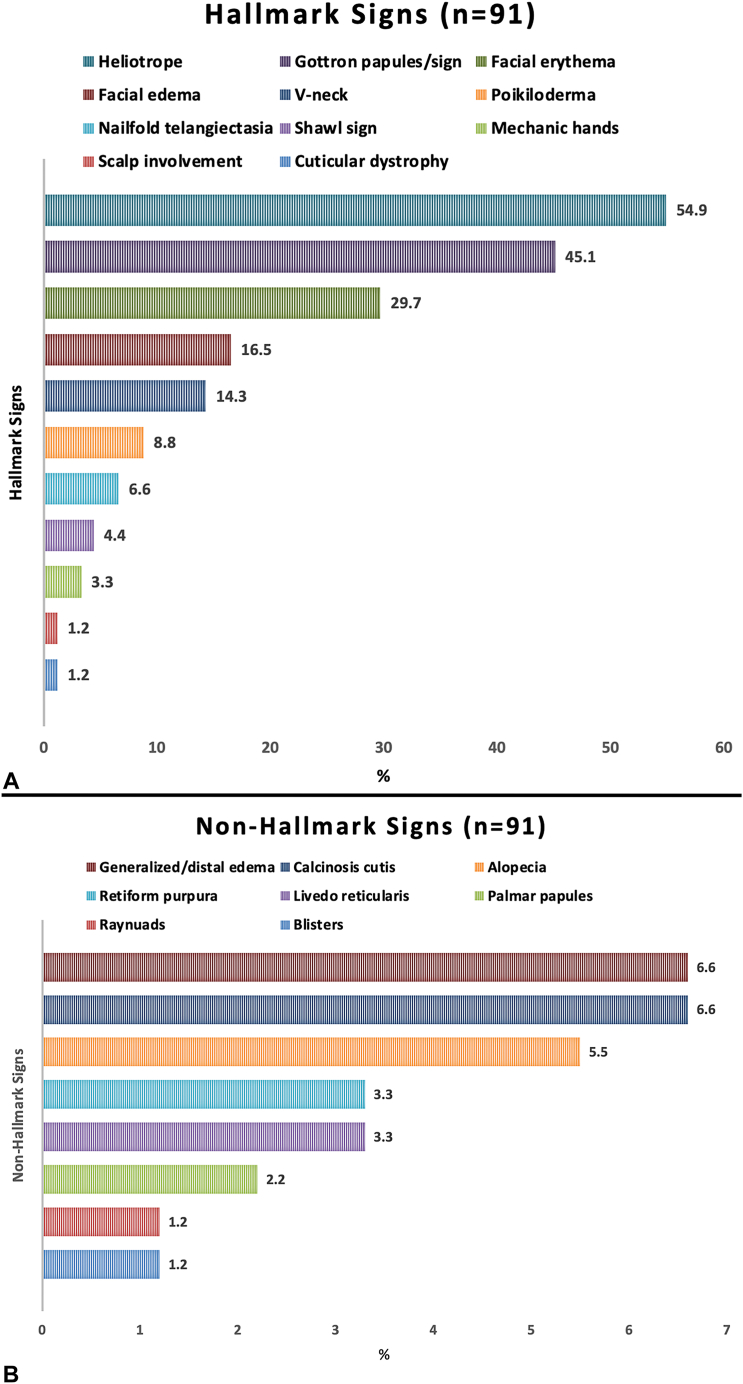
Hallmark (A) and nonhallmark (B) features in patients with dermatomyositis-associated panniculitis. *DMS*, Dermatomyositis.

Of 75 patients (information available), 72% (*n* = 54) had ADMS and 28% (*n* = 21) JDMS (adult:juvenile ratio of ∼2.6:1). Females predominated in both categories (ADMS 6.7:1; JDMS 3.2:1). Regarding muscle disease (available, *n* = 89), most (91%, *n* = 81) were myopathic and 9% (*n* = 8) amyopathic. Overall (*n* = 91), 54.9% (*n* = 50) reported a heliotrope rash, 45.1% (*n* = 41) Gottron papules/sign, 29.7% (*n* = 27) facial erythema, 16.5% (*n* = 15) facial edema, 14.3% (*n* = 13) V-neck, 8.8% (*n* = 8) poikiloderma, 6.6% (*n* = 6) nail fold telangiectasia, 4.4% (*n* = 4) shawl, 3.3% (*n* = 3) mechanic hands, while 1.2% (*n* = 1) each reported cuticular dystrophy and scalp involvement. Nonhallmark signs were reported in 35 cases; 6.6% (*n* = 6) with generalized/distal edema, 6.6% (*n* = 6) clinical calcinosis cutis, 5.5% (*n* = 5) alopecia, 3.3% each (*n* = 3) livedo reticularis/retiform purpura, 2.2% (*n* = 2) palmar papules, and 1.2% (*n* = 1) each reported Raynaud phenomenon and blisters. Overall, most patients with panniculitis had myopathic adult dermatomyositis with hallmark signs (Fig 2, *A* and *B*).

### Panniculitis: Clinical features

**Fig 3:**
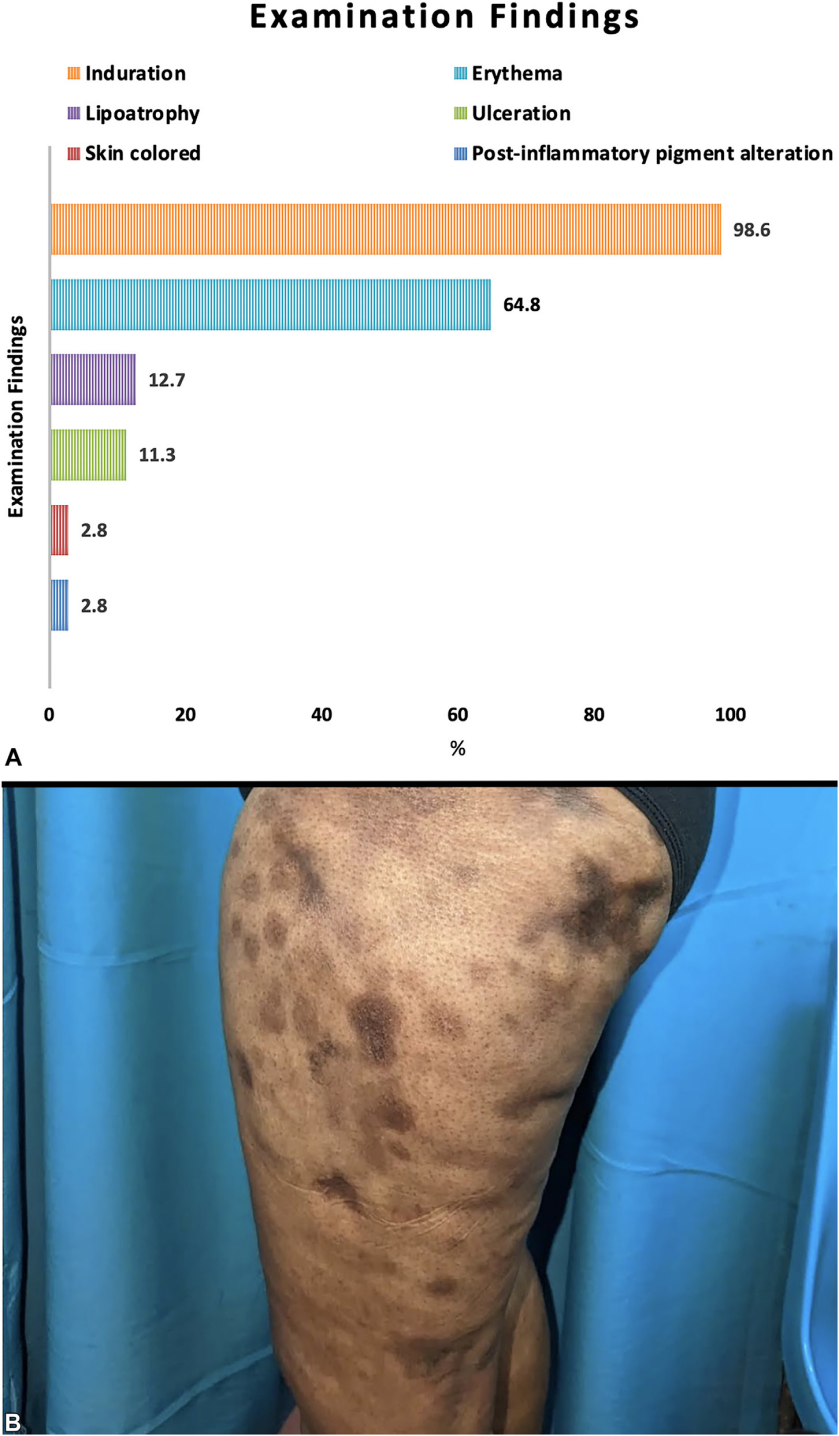
Reported examination findings and clinical appearance of dermatomyositis-associated panniculitis. Indurated erythematous nodules/plaques predominate in dermatomyositis-associated panniculitis, but pigment alternation and lipoatrophy similar to lupus panniculitis are also reported (**A**). Note the clinical similarity to lupus panniculitis in this this young woman with dermatomyositis-associated panniculitis and hyperpigmented indurated plaques/nodules with associated lipoatrophy involving her thighs and buttock (**B**).

Panniculitis onset relative to diagnostic DMS features was evaluable for 63 cases. Of these, 66.7% (*n* = 42) reported DMS prior to panniculitis, 17.5% (*n* = 11) had panniculitis predating classical DMS features, and in 15.9% (*n* = 10) panniculitis/classical DMS occurred simultaneously. Symptoms (pain/tenderness) occurred in 53.8%, (*n* = 49). Seventy-one cases reported examination findings (Fig 3), 98.6% (*n* = 70) with indurated plaques/nodules. Most (64.8%, *n* = 46) were erythematous, 2.8% (*n* = 2) skin-colored, and 2.8% exhibited postinflammatory pigment alteration. Focal lipoatrophy was seen in 12.7% (*n* = 9) and 11.3% (*n* = 8) ulcerated.

**Fig 4:**
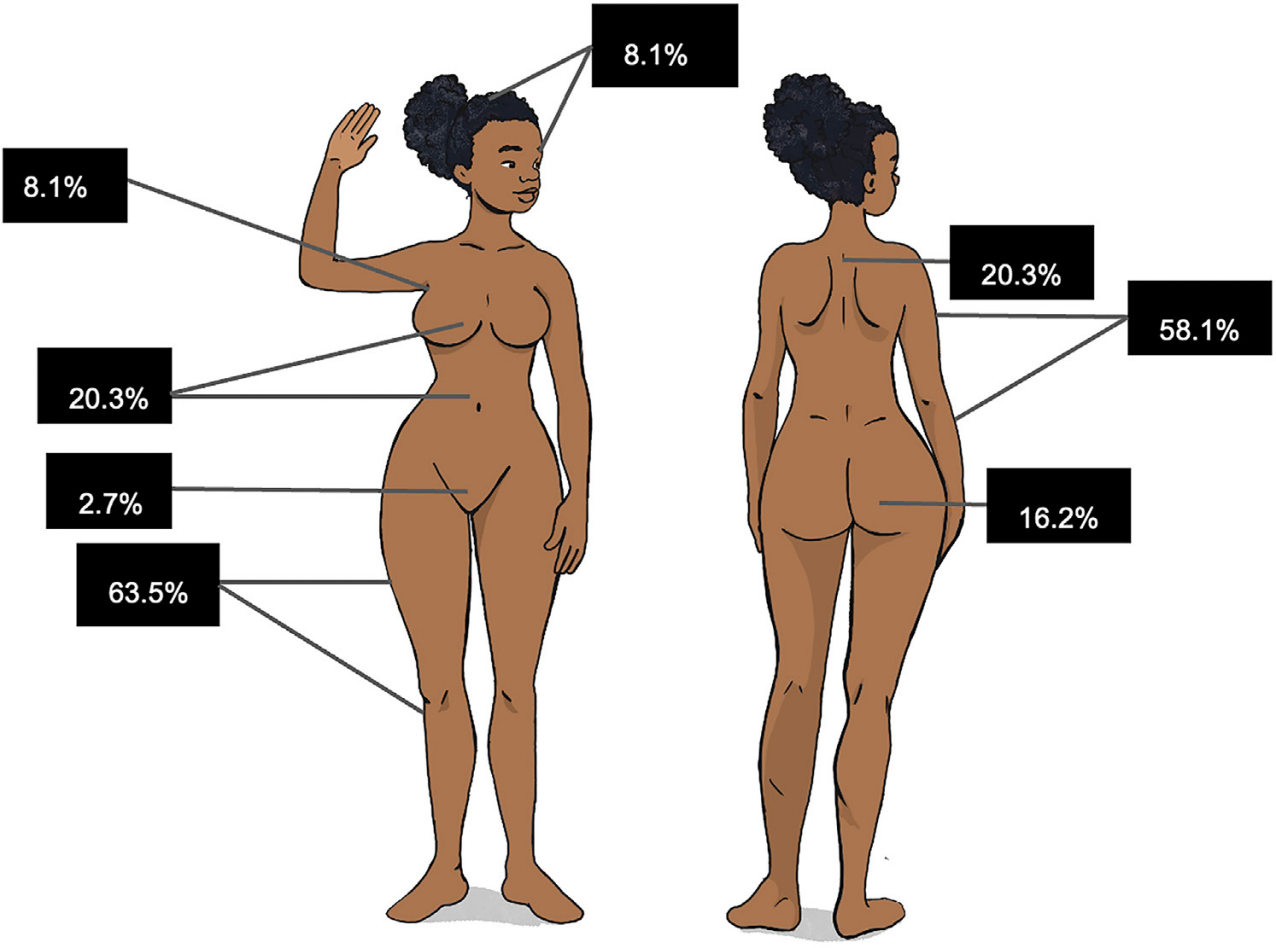
Overall distribution of dermatomyositis-associated panniculitis. This figure represents the overall involvement by panniculitis. An individual may have multiple areas involved.

Regarding location (*n* = 74), overall, 50% (*n* = 37) had lesions at multiple sites. For single-location involvement, lower limbs were most commonly affected (25.6%, *n* = 19), then upper limbs (14.9%, *n* = 11), face/scalp (4.1%, *n* = 3), buttock (2.7%, *n* = 2), torso (1.4%, *n* = 1), and a single patient had mesenteric panniculitis. In mixed cases (*n* = 37), 86.5% (*n* = 32) had upper limb involvement in some capacity, lower limbs in 75.7% (*n* = 28), torso in 35.1% (*n* = 13), buttocks in 27% (*n* = 10), axilla in 16.2% (*n* = 6), face in 8.1% (*n* = 3), and genitals in 5.4% (*n* = 2). Globally (Fig 4), 63.5% (*n* = 47) had some involvement of the lower limbs, 58.1% (*n* = 43) the upper limbs, 20.3% (*n* = 15) the torso, 16.2% (*n* = 12) the buttocks, 8.1% (*n* = 6) each the axilla and face/scalp and genital in 2.7% (*n* = 2).

### Triggering/etiologic factors

Most cases had no clear triggering/etiologic factor; 2.2% (*n* = 2) suspected to be drug-related (methotrexate and minocycline).^20^^,^^29^

### Extracutaneous manifestations

Overall (*n* = 91), 20.9% (*n* = 19) had ≧1 extracutaneous manifestation (Table I). Of these, interstitial lung/pulmonary disease predominated (68.4%, *n* = 13), then arthritis/arthralgia (47.4%, *n* = 9), gastrointestinal complications (21.1%, *n* = 4 [intestinal perforation/dysphagia/hepatomegaly]), and cardiac (pericardial effusion, each 5.3%, *n* = 1). Malignancy was reported in 7.4% (*n* = 4) of adult cases (ovarian, breast, nasopharyngeal carcinoma, and rhabdomyosarcoma).^8^^,^^12^^,^^57^^,^^59^

**Table I:** Extracutaneous manifestations, antibody profiles, and prognosis reported in patients with dermatomyositis-associated panniculitis

Extracutaneous features (*n* = 19) % (*n*)	Antibody profiles % (*n*)	Prognosis (*n* = 49) % (*n*)
*Pulmonary/ILD* 68.4 (*n* = 13)	DMS-associated *Anti-MDA5:* 13.2 (*n* = 12) *Anti-Mi-2*: 7.8 (*n* = 7) *Anti-TIF1-γ:* 7.8 (*n* = 7) Anti-NXP2: 3.3 (*n* = 3) Anti-SAE: 1.1 (*n* = 1)	*Good/complete response* 67.3 (*n* = 33)
*Joint involvement* 47.4 (*n* = 9)		*Partial response* 10.2 (*n* = 5)
*Gastrointestinal* 21.1 (*n* = 4)		*Poor response* 22.4 (*n* = 11)
	Antinuclear antibodies *ANA*: 35.2 (*n* = 32) *Anti-SSA:* 3.3 (*n* = 3) *Anti-dsDNA:* 2.2 (*n* = 2) *Anticentromere:* 1.1 (*n* = 1) *Anti-ribonucleoprotein:* 1.1 (*n* = 1)	
*Cardiac* 5.3 (*n* = 1)		
*Malignancy (in ADMS)* 7.4 (*n* = 4)		

*AMDS*, Adult dermatomyositis; *ANA*, antinuclear antibody; *DMS*, dermatomyositis; *dsDNA*, double stranded deoxyribonucleic acid; *ILD*, interstitial lung disease; *MDA5*, melanoma differentiation-associated protein 5; *NXP2*, nuclear matrix protein 2; *SSA*, steroid sparing agent; *TIF1*, transcriptional intermediary factor 1.

### Autoantibody profile

Positive antinuclear antibodies were reported in 35.2% (*n* = 32); antisteroid sparing agent in 3.3% (*n* = 3), double stranded DNA in 2.2% (*n* = 2), and anticentromere and anti-ribonucleoprotein antibodies in 1.1% (*n* = 1) each. DMS-specific/associated antibodies were recorded in 33% (*n* = 30). In decreasing frequency were antibodies against MDA5 (*n* = 12), Mi-2 (*n* = 7), transcriptional intermediary factor 1-γ (*n* = 7), nuclear matrix protein 2 (*n* = 3), and SUMO-activating enzyme subunit 1 (*n* = 1). Seven anti-MDA5 positive cases had interstitial lung disease and one, rhabdomyosarcoma. See Table I.

### Histopathology

Seventy-four cases detailed specific histopathologic findings (Table II); 54.1% (*n* = 40) were lobular, 29.7% (*n* = 22) mixed but lobular predominant, 5.4% (*n* = 4) septal, 1.4% (*n* = 1) mixed but septal predominant, and 6.8% (*n* = 5) mixed without clear septal/lobular predominance. Two cases documented panniculitic inflammation without further detail; 40.5% (*n* = 30) had a lymphoplasmacytic infiltrate, 16.2% (*n* = 12) a lymphocytic/lymphohistiocytic, 5.4% (*n* = 4) neutrophil-predominant, and 4.1% (*n* = 3) mixed neutrophil-lymphocytic/lymphoplasmacytic. Histiocyte-predominant/granulomatous inflammation was present in 2.7% (*n* = 2). The remainder lacked inflammatory cell composition details; 29.7% (*n* = 22) reported fat necrosis, 28.4% (*n* = 21) calcium, 25.7% (*n* = 19) hyalinization, 14.9% (*n* = 11) lymphocytic rimming of adipocytes, 14.9% (*n* = 11) lipomembranous change, 8.1% (*n* = 6) each lymphocytic vasculitis, lymphocytic karyorrhexis, and thrombosis/vasculopathy, and 6.8% (*n* = 5) lymphoid follicles. Regarding epidermal/dermal changes: 28.4% (*n* = 21) reported increased dermal mucin, 25.7% (*n* = 19) interface dermatitis, and 20.3% (*n* = 15) perivascular and/or periadnexal lymphocytic infiltrates. Follicular plugging and ulceration were each reported in 1.4% (*n* = 1). The predominant pathologic finding in PDMS was a lymphocytic/lymphoplasmacytic lobular/lobular-predominant panniculitis with/without overlying epidermal/dermal changes typical of AICTDs.

**Table II:** Reported histopathologic features in patients with dermatomyositis-associated panniculitis

Panniculitis pattern (%/*n*)		Cell type (%/*n*)		Epidermal/dermal features (%/*n*)		Additional subcutaneous features (%/*n*)	
Lobular	54.1 (*n* = 40)	Lymphoplasmacytic	40.5 (*n* = 30)	Increased dermal mucin	28.4 (*n* = 21)	Calcium	28.4 (*n* = 21)
Mixed (lobular predominant)	29.7 (*n* = 22)	Lymphocytic/lympho-histiocytic	16.2 (*n* = 12)	Interface dermatitis	25.7 (*n* = 19)	Thrombosis/vasculopathy	8.1 (*n* = 6)
Septal	5.4 (*n* = 4)	Neutrophilic	5.4 (*n* = 4)	PVPALI	20.3 (*n* = 15)	Lymphoid follicles	6.8 (*n* = 5)
Mixed (septal predominant)	1.4 (*n* = 1)	Mixed neutrophilic lymphocytic/lymphoplasmacytic	4.1 (*n* = 3)	Follicular plugging	1.4 (*n* = 1)	Lymphocytic vasculitis	8.1 (*n* = 6)
Mixed without clear septal/lobular predominance	6.8 (*n* = 5)	Histiocytic/granulomatous	2.7 (*n* = 2)			Lipomembranous change	14.9 (*n* = 11)
				Ulceration	1.4 (*n* = 1)	Hyalinization	25.7 (*n* = 19)
						Lymphocytic karyorrhexis	8.1 (*n* = 6)
						Fat necrosis	29.7 (*n* = 22)
						Lymphocytic rimming of adipocytes	14.9 (*n* = 11)

*PVPALI*, Perivascular and/or periadnexal lymphocytic infiltrate.

### Treatment and prognosis

Treatment details were available in 54 cases. In 50% (*n* = 27), an additional/specific dermatomyositis treatment (SDMS) was started/added to GDMS to achieve control. In the remaining 50%, treatment was included in GDMS flare management often with a dose adjustment of the preexisting regimen. For GDMS, 48% (*n* = 26/54) received a systemic glucocorticoid (GC) and a steroid sparing agent (SSA), 22.2% (*n* = 12) a GC alone, 18.5% (*n* = 11) combined GC, steroid sparing, and biologic (IVIG or rituximab), 7.4% (*n* = 4) a SSA only, and 1.9% (*n* = 1) a biologic only. The SSA/biologics used in various combinations and arranged in order of frequency included: methotrexate (*n* = 16), azathioprine (*n* = 14), antimalarials (*n* = 9), IVIG (*n* = 9), calcineurin inhibitor (tacrolimus or cyclosporine, *n* = 9), cyclophosphamide (*n* = 4), rituximab (*n* = 3), and mycophenolate mofetil, dapsone, colchicine, thalidomide, pentoxifylline, chlorambucil, and salicylate of soda (*n* = 1 each). Most SDMS interventions involved multiagent rescue. When disaggregated, as single agents or in combination, specific therapies used in order of frequency included the following: antimalarials (*n* = 9), methotrexate (*n* = 7), IVIG (*n* = 4), GC (*n* = 5), rituximab (*n* = 2), cyclosporine (*n* = 4), JAK inhibitors (*n* = 2), azathioprine (*n* = 2) and dapsone, colchicine, chlorambucil, diclofenac, intralesional triamcinolone, and antireticulocyte cytotoxic serum (*n* = 1 each).

Prognosis was available in 49. Most (67.3%, *n* = 33) had a good/complete response, 10.2% (*n* = 5) a partial response and 22.4% (*n* = 11) a poor response. Three patients died (cardiac complications, pneumonia and intestinal vasculopathy). Overall, therapy most often included a GC and SSA. The 5 most frequently used non-GC therapies arranged in order of frequency included methotrexate, antimalarials, azathioprine, IVIG, and calcineurin inhibitors. Patients with PDMS generally have a good response to therapy, but treatment resistant disease exists.

## Discussion

The most typical patient with PDMS is a young adult female with classical myopathic DMS. Panniculitis occurred across the first to ninth decades of life but was 3 times more common in adults. Both ADMS and JDMS had an increased F:M ratio, although less pronounced in children. The female preponderance may represent the overall skewed F:M ratio in DMS/AICTDs.^61^^,^^62^ Perhaps relative increased female body fat percentages and typical fat distribution patterns (more pronounced in adults but present in childhood) contribute to overrepresentation.^63^^,^^64^ While racial/ethnic information was only available for just over ⅓ of patients, where evaluable, Asian persons accounted for ∼¾, and, reports published from Asia (relatively racially homogeneous populations)^65^ for ∼⅓ of all studies. This may suggest predilection, although caveats include relative underreporting from countries with high proportions of Black persons and global/regional variation in race reporting in case reports.^66^

Patients demonstrated the gamut of classical cutaneous DMS features, most commonly facial signs and Gottron papules/sign. Nonhallmark signs were less common. PDMS associated with muscle involvement and amyopathic disease was less frequent (9%). Variation occurred in temporal association of panniculitis with the appearance of reliably diagnostic DMS features, ranging from 6 years prior to 32 years after.^4^^,^^38^ Recognition that >10% of PDMS occurs before diagnostic criteria for DMS is important. Given the overlapping clinicopathologic features with lupus panniculitis (LP) (below), clinicians should keep evolving DMS in their differential list for AICTD-related panniculitis.

DMS-panniculitis closely mimics LP (Fig 3, *B*). Like LP, painful, indurated nodules involving the limbs of women in the fourth decade of life are the most common presentation.^1^ Unusual locations included axilla (∼8%) and genitals (∼3%). One apparent difference is relatively low rates of residual lipoatrophy (12.7%), seen in >60% of LP.^1^ As most occurred after/simultaneously with myopathic DMS, more aggressive or preexisting immunosuppression might account for less residual lipoatrophy.

Less than ¼ of patients with PDMS had extracutaneous disease. Nevertheless, systemic evaluation (particularly for interstitial lung disease and arthritis/arthralgia) should be undertaken. Abdominal pain may indicate mesenteric panniculitis or intestinal perforation.^22^ While all adults should be screened, cancer appears uncommonly associated with PDMS (7.4%). Immunologically, antinuclear antibody positivity may be seen in at least ⅓ of patients and again, embrace caution when encountering a patient with autoimmune connective tissue disease-panniculitis and a positive antinuclear antibody. As LP is more common, it will often represent the correct diagnosis, nevertheless, clinicians should reserve room for considering DMS. Approximately ⅓ reported positive myositis-associated/specific antibodies, although many reports did not test these. Anti-MDA5 antibodies predominated (*n* = 12). Although too few reported profiles to be conclusive, anti-MDA5 antibodies may be overrepresented, being rare in many populations (7%-16%).^67^ Could the relative proportion of reports from Asia where anti-MDA5 may occur in up to 60% of cases contribute to potential predominance?^68^

Diagnosis hinges on histopathology although imaging may demonstrate disease.^22^^,^^28^ While indurated nodules should prompt consideration of panniculitis, biopsy is recommended. In our literature review, we encountered 6 cases of infectious panniculitis (*Histoplasma sp*. [*n* = 4] and *Staphylococcus aureus* [*n* = 2]) with clinical features indistinguishable from AICTD-related panniculitis, imparting potentially devastating consequences with immunosuppression escalation.^69^, ^70^, ^71^, ^72^ All infectious cases had a preexisting DMS diagnosis on immunosuppressants contributing to opportunistic infection. Additionally, although beyond the study scope, in our excluded papers, we encountered cases with similar clinical presentations representing subcutaneous lymphomas, diagnosed only with histopathology.^73^, ^74^, ^75^, ^76^

In most PDMS, a lobular/lobular-predominant panniculitis similar to LP was observed. A lympoplasmaytic/lymphocytic infiltrate was most commonly seen, although histiocytic/granulomatous and neutrophilic infiltrates also occurred. Special stains/tissue cultures are prudent in neutrophil/histiocytic-rich cases to exclude infectious panniculitis. Other LP-like changes including lymphocytic adipocyte rimming, hyalinization, lymphoid follicles, thrombosis, and lymphocytic vasculitis were observed in some. Overlying epidermal/dermal changes (lupus profundus-like) were reported in ∼¼ of patients. There were no reliable histopathologic differences between lupus and DMS-associated panniculitis. We did note, however, wider possible findings in DMS. Specifically, 6.8% of cases had septal/septal-predominant panniculitis, more commonly seen in entities like erythema nodosum.

Whether during G/SDMS treatment, multiagent immunosuppression/immunomodulation appears necessary. Systemic GC combined with a variety of SSA and/or biologics were employed. A specific regimen cannot currently be recommended. Popular SSAs included various combinations of methotrexate, antimalarials, azathioprine, IVIG, and calcineurin inhibitors. We noted success of IVIG in unresponsive cases, although no significant association between IVIG use and prognosis was found (too few cases; *P = .59*). Interestingly, successful JAK-inhibitor rescue was described (*n* = 2), implying an emerging role for these agents.^6^^,^^35^ Fortunately, good/complete response was common, although multiregimen resistant disease was reported (22.4%).

In summary, we describe clinicopathologic features, management, and prognosis of panniculitis in patients with DMS. Most are young females with clinical and histopathologic features similar to LP. Multiagent immunosuppression induces a clinical response in most. Limitations include the retrospective nature, inconsistent reporting of parameters and lack of longitudinal data.

## Conflicts of interest

None disclosed.
